# Phytochemicals profiling of *Cassia fistula* fruit extract and its effect on serum lipids and hematological parameters in high‐fat diet‐induced hyperlipidemic female rats

**DOI:** 10.1002/fsn3.4229

**Published:** 2024-05-21

**Authors:** Maryam Tariq, Nazir Ahmad, Mahr Un Nisa, Muhammad Abdul Rahim, Eliasse Zongo

**Affiliations:** ^1^ Department of Nutritional Sciences Government College University Faisalabad Faisalabad Pakistan; ^2^ Department of Food Science, Faculty of Life Sciences Government College University Faisalabad Pakistan; ^3^ Department of Food Science & Nutrition, Faculty of Medicine and Allied Health Sciences Times Institute Multan Pakistan; ^4^ Laboratoire de Recherche et d'Enseignement en Santé et Biotechnologies Animales Université Nazi BONI Bobo Dioulasso Burkina Faso

**Keywords:** *C. fistula* fruit, extract, hyperlipidemia, total phenolic contents, UAE

## Abstract

*Cassia fistula* (*C. fistula*) has shown strong anti‐inflammatory, hepatoprotective, antitussive, antibacterial, and antifungal properties and is being used for healing wounds and gastrointestinal illness. This study was planned to obtain fruit extract from *C. fistula* using ultrasonic‐assisted extraction (UAE) technique and evaluated for phytochemical contents, anti‐hyperlipidemia, and hematological parameters. The results showed that total phenolic (TPC), total flavonoids (TFC), condensed tannin (CT), and saponins were 13.07 mg GAE/g, 5.24 mg QE/g, 4.01 mg/g, and 27.55%, respectively, in the extract. Proximate composition of the extract showed 2.48%, moisture, 1.25% fat, 2.80% ash, 4.59% fiber, 11.93% protein, and 76.95% NFE. The 2, 2‐diphenyl‐1‐picrylhydrazyl (DPPH) and ferric reducing antioxidant power assay (FRAP) activity was 63.30 μg/mL and 15.02 nmol/g, respectively. High‐fat diet (HFD)‐induced hyperlipidemic rats were orally administrated with 0.5 and 1.0 g of extract/kg body weight (bw) daily. The reduction of total cholesterol (TC: 90.83 ± 8.86 mg/dL), triglycerides (TG; 74.16 ± 9.10 mg/dL), and low‐density lipoproteins (LDL; 74.83 ± 4.66 mg/dL) and increase of high‐density lipoproteins (HDL; 41.83 ± 8.4 mg/dL) was observed. Significant changes in red blood cells (RBCs; 8.03 ± 0.6710^6^/μL), mean corpuscular hemoglobin concentration (MCHC; 35.02 ± 1.78 g/dL), mean corpuscular hemoglobin (MCH; 18.00 ± 0.26 pg), and mean corpuscular volume (MCV; 55.36 ± 4.01 fL) at 1.0 g extract intake was observed. Extract administration also improved significantly liver enzymes, body weight, and liver morphology. Therefore, *C. fistula* extract can be effectively used as a therapeutic agent to improve serum biochemistry and hematological values.

## Graphical abstract

The purpose of the current research was to extract and evaluate the therapeutic potential of natural *C. fistula* pod extract against hyperlipidemia.

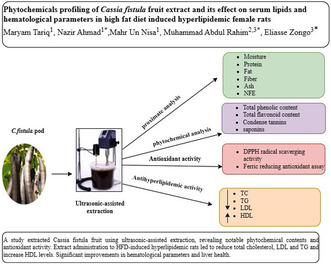

## 1 INTRODUCTION

Hyperlipidemia is a metabolic disorder characterized by abnormal blood lipid levels, resulting in damage of endothelial bloody vessels (Fioranelli et al., 2018; Jaffe & Karumanchi, 2024; Rafaqat et al., 2024). This damage leads to a loss of nitric oxide in an affected region, ultimately escalating an inflammatory response and promoting lipid accumulation in an endothelial wall and leading to a formation of foam cells. Subsequently, activated macrophages engulf these foam cells and result in development of atherosclerosis, necrosis, mitochondrial dysfunction, and apoptosis (Cao et al., 2017; Howell et al., 2011; Naser et al., 2021). According to WHO, a global prevalence of hyperlipidemia is estimated to be 37% and 40% in males and females, respectively. Notably, 98.1% and 97.3% of newly diagnosed diabetics and pre‐diabetic individuals exhibited dyslipidemia, while prevalence in non‐diabetic Pakistani population is around 95.2% (Alshamiri et al., 2018; Basit et al., 2020).

Current medicants used for lipid regulation can have significant side effects, necessitating an exploration of alternative strategies. Natural treatments offer a promising avenue for reducing hyperlipidemia risks (Chattopadhyaya et al., 1996; Krause & Newton, 1995). Phenolic acids, flavonoids, epiafzelechin, epicatechin, saponins, procyanidin, proanthocyanidins, and anthocyanins have strong potential to regulate blood lipids (Nagpal et al., 2011). The dietary intake of 5%–10% polyphenols, flavonoids, and anthocyanins of diet for 21 days significantly regulated dyslipidemia in rats fed on high‐cholesterol diet (Queiroz et al., 2017). Oral intake of a polyphenol‐rich extract of 0.47 and 1.87 g/kg bw for 4 weeks reduced serum TG in HFD‐induced diabetic rats (Li et al., 2015). Flavonoids at a dose of 10 mg/kg bw per day also inhibited cholesterogenesis in atherogenic animals (Park et al., 2015). Procyanidins intake of 0.1 g/kg bw on a daily basis for 24 weeks offered positive effects on atherosclerosis and hyperlipidemia in HFD‐fed mice through lipid metabolic and liver cholesterol synthesis (Rong et al., 2017).

Thus, plants rich in phytochemicals have been used since ancient times for treatment of various diseases. *C. fistula* is one of the trees that has been traditionally used for therapeutic purposes. Its barks, leaves, and fruits (pods) have been used for many diseases. *C. fistula* pod contains a high amount of epiafzelechin, epicatechin, procyanidin, and saponins and can have protective role in metabolic disorders (Kashiwada et al., 1990; Mwangi et al., 2021). Therefore, the current research aimed to evaluate the phytochemical profile and therapeutic potential of *C. fistula* pod extract in hyperlipidemia.

## 2 METHODS AND MATERIALS

### 2.1 Procurement of raw material

*Cassia fistula* pods were procured from the local market, cleaned, and dried under sunlight. The pods were ground in a laboratory mill and stored for further analysis.

### 2.2 Extract preparation

A 500 g powder of *C. fistula* pods was immersed in 200 mL distilled water and was left for the night. Then, it was boiled for 3–4 h and further extraction occurred by using ultrasonic‐assisted extraction (UAE) method accordingly (Yingngam et al., 2019). For this purpose, the mixture was placed in a 400 mL beaker, placed in an ultrasonic water bath (Branson 3210, USA), and subjected to UAE treatment for 20 min at 45°C and 20% of amplitude. The decoction was cooled for 30 min at room temperature and then filtered through muslin cloth and further concentrated using a rotary evaporator at 50°C (Galviz‐Quezada et al., 2019).

### 2.3 Proximate analysis

The proximate analysis was performed using Association of Official Analytical Chemists (AOAC, 2005). Total nitrogen content was determined by the Kjeldahl method (Method# 976.05) and protein was calculated by using a factor of 6.25. Soxhlet method (Method# 920.39) was used for crude fat. Crude fiber was obtained after sample digestion (Method 962.09). Moisture was calculated after oven drying at 60°C for 24 h (Method 925.10). Ash was determined after heating at 550°C till ashing (Method 942.05).

### 2.4 Determination of phytochemicals

#### 2.4.1 Total phenolic contents (TPC)

Folin–Ciocalteu was used for TPC accordingly (Singleton et al., 1999). The sample (0.5 mL) was poured into a tube containing 0.1 mL of Folin–Ciocalteu reagent. Then, 1.0 mL (7%) Na_2_CO_3_ was added and incubated for 1 h. The absorbance was recorded at 750 nm. A standard solution was prepared by dissolving 3 mg of gallic acid and a series of dilutions was made by using distilled water. Results are presented as mg gallic acid/g dry weight sample.

#### 2.4.2 Total flavonoid content

Total flavonoid content (TFC) were determined using the method of Aiyegoro and Okoh (2010) with small modifications. Standard solution was prepared by dissolving 10 mg quercetin in methanol and dilutions (0, 2, 4, 6, 8, 10 μg/mL) were prepared to obtain the standard curve. A 1.0 mL sample, 4 mL of distilled water, and 0.3 mL of a 5% NaNO2 solution were mixed and incubated for 10 min. Following a 10‐min interval, 0.3 mL of a 10% AlCl3 solution was added. Subsequently, 2 mL of a 1.0 M NaOH solution was incorporated and making a total volume of 10 mL and absorbance was measured by UV spectrophotometer. TFCs were calculated from standard curve extrapolation and expressed as mg quercetin/g dry weight sample.

#### 2.4.3 Condense tannin content (CT)

For CT contents determination, 0.5 mL of sample, 3 mL of vanillin solution (4%), and 1.5 mL of HCl were mixed for 10 min. The absorbance was recorded at 500 nm (Lister & Wilson, 2001). CT contents were measured with gallic acid standard and the results were expressed as mg gallic acid/g dry weight sample.

#### 2.4.4 Saponins extraction/determination

The extraction/determination of saponins was determined through the procedure of Mora‐Ocación et al. (2022). First, 20 g of the sample was defatted with 300 mL petroleum ether and then was air‐dried. A volume of 300 mL methanol was used for the extraction of saponins from a sample that was kept overnight, and then 200 mL of water‐butanol (1:1 v/v) was mixed with methanol extract. It was further extracted with 50 mL of acetone to obtain saponins. A volume of 1.0 mL of standard solution and 3.5 mL of Liebermann‐Burchard reagent was used for optimal wavelength determination. Standard dilutions (0–0.4 mg/mL) and 1.0 mL of reagent were mixed and incubated for 30 min and absorbance was measured.

### 2.5 Antioxidant activity

DPPH and FRAP assay were used to determine the antioxidant activity of *C. fistula* extract.

#### 2.5.1 DPPH assay

To prepare DPPH solution, 7.89 mg of DPPH was dissolved in 100 mL of absolute ethanol. The solution was then stored in darkness for 2 h. The 1.0 mL DPPH solution and 0.8 mL Tris–HCl were mixed and then 0.2 mL sample solution was added and incubated for 30 min at room temperature. The absorbance at 517 nm was recorded. Blank reading was taken using only 1.2 mL of ethanol and 0.8 mL of Tris–HCl Results were expressed as EC50 DPPH activity. EC50 was determined by plotting inhibition ratios (*y*) against sample concentrations (x) at six data points and fitting a regression line (*y* = ax + b). EC50 value was calculated by interpolating between two points where inhibition reached approximately 50%, and drawing a straight line to achieve 50% inhibition at the intersection point, forming another regression line (*Y* = AX + B). The concentration of the sample (X) corresponds to 50% inhibition using equation *Y* = AX + B (Valko et al., 2007). The inhibition ratio was calculated as:

$$\begin{array}{c} \text{DPPH}\;\left(\%\text{inhibition}\right)=\\ \left[\left(\text{control absorbance}-\text{sample absorbance}\right)/\text{control absorbance}\right]\times 100 \end{array}$$

#### 2.5.2 FRAP assay

The FRAP activity was measured using Benzie and Strain (1996) method. To prepare the FRAP solution, 10 mM TPTZ and 20 mM ferric chloride were combined in a 0.25 M acetate buffer with a pH of 3.6. Then, 0.15 mL of sample was mixed with 400 μL of water, and after 5 min, 3 mL of FRAP reagent was mixed. The absorbance was taken at 593 nm and the results were calculated as nmol Fe^2+^ equivalent/g dry extract.

## 3 EXPERIMENTAL DESIGN

Twenty‐four healthy female rats were weighed 190–200 g and kept in an animal room of the facility of the Department of Physiology, Government College University Faisalabad. Rats were caged individually with proper clean bedding and controlled with specific temperature and light. The whole research was conducted according to the ethical guidelines of the laboratory animal care (Gonder & Laber, 2007).

### 3.1 High‐fat diet (HFD) and induction of hyperlipidemia

Induction of hyperlipidemia in experimental animals was done using HFD {fat (58%), carbohydrate (17%), protein (25%) of total kcal} according to the procedure of Jain and Surana (2016) for 30 days. Induction was verified through lipid profile according to the criteria of “National Cholesterol Education Program Expert Panel on Detection, Evaluation, and Treatment of High Blood Cholesterol in Adults (Adult Treatment Panel III) (2001).” While control group rats were given a standard diet along with ad libitum throughout the trial.

### 3.2 Treatment plan

Rats were divided into four groups and each group contained six rats and *C. fistula* extract administration plan is shown in Table 1. *C. fistula* extract doses of 0.5 and 1.0 g /kg body weight were administrated through gavage. The doses were decided from a previous study (Abid et al., 2016). The intervention was carried out for a period of 1 month.

**TABLE 1:** Treatment plan.

Group 1	Not induced rats	Standard diet
Group 2	Induced rats	Standard diet
Group 3	Induced rats	Standard diet + 0.5 g extract
Group 4	Induced rats	Standard diet + 1.0 g extract

### 3.3 Data collection

The data was collected for the following parameters during the study.

#### 3.3.1 Feed and water intake

The food intake of experimental rats was calculated on a daily basis by subtracting leftovers from the total diet offered and water was given through graduated drinking bottles and its intake was measured on a daily basis (Ahmad et al., 2018).

#### 3.3.2 Body weight

The body weight of rats was measured on a weekly basis to determine the impact of treatment on body weight by using a digital weighing scale (QERINKLE).

#### 3.3.3 Collection of blood sample

After the end of the trial, overnight fasting rats were decapitated, and blood samples were taken in a yellow‐coated tube. Centrifuged at RCF (g): 1252 for 10 min (SCILOGEX DM0636, Model # 91502303999) and serum was separated and frozen for further analysis.

#### 3.3.4 Serum lipid analysis

Total cholesterol (TC) and TG were analyzed by enzymatic method following the modified protocol of Kim et al. (2011). HDL and LDL were assessed by the enzymatic method followed by the modifying protocol of Alshatwi et al. (2010).

#### 3.3.5 Hepatic safety test

Aspartate aminotransferase (AST) and alanine aminotransferase (ALT) were measured by Sigma Aldrich Kit. Both tests were performed according to the instructions mentioned by the manufacturer.

#### 3.3.6 Hematological parameters

Blood samples were collected and used to analyze the hematological parameters such as Red blood cell (RBC), mean corpuscular hemoglobin (MCH), mean corpuscular hemoglobin concentration (MCHC), platelets, monocytes, and neutrophil, hemoglobin (Hb), using Biochem analyzer (Mindray Auto Hematology Analyzer, BC‐5200, USA).

#### 3.3.7 Histopathological study

Liver samples were fixed in 10% formalin, followed by embedding in paraffin and sectioning to a thickness of 5 mm for histological analysis. These sections were then stained with hematoxylin and eosin for further examination. Pathological images were recorded at 10× magnification using a light microscope (Model BA410E Carlsbad, USA). It was evaluated using the method of Bernet et al. (1999).

### 3.4 Statistical analysis

Triplicate data in an Excel sheet was used for data calculation. Mean ± standard deviation results are presented. One‐way ANOVA (Statistix version 10) and the least significant difference (LSD) test were used to examine group differences. The *p* < .05 was used as significant.

## 4 RESULTS AND DISCUSSION

### 4.1 Extraction and physiochemical and antioxidant characterization

The UAE extraction, proximate analysis, phytochemicals analysis, and antioxidant activity results are presented in Table 2. The yield of extract from *C. fistula* pods was 25 ± 0.04%. The extract on a dry basis had 2.48 ± 0.15% moisture, 1.25 ± 0.55% fat, 11.93 ± 1.55% protein, 4.59 ± 0.55% fiber, 2.80 ± 0.20% ash, and 76.95 ± 0.55% NFE, respectively. The amount of TPC, TFC, CT, and saponins in the extract were 13.07 + 0.14 mg/g, 5.24 + 0.02 mg QE/g, 4.01 ± 0.52 mg/g, and 27.55 ± 0.59%, respectively. The antioxidant activity of DPPH and FRAP of the extract were 63.30 ± 0.015 μg/mL and 15.02 ± 0.34 nmol/g, respectively.

**TABLE 2:** Compositional and physiochemical analysis of *C. fistula* extract.

Physiochemical analysis	Parameters	Plant extract
UAE extraction	Yield	25 ± 0.04%
Proximate analysis	Moisture (%)	2.48 ± 0.15
	Fiber (%)	4.59 ± 0.55
	Protein (%)	11.93 ± 1.55
	Ash (%)	2.80 + 0.20
	Fat (%)	1.25 ± 0.55
	NEF (%)	76.95 ± 0.50
Phytochemicals analysis	TPC (mg GAE/g)	13.07 ± 0.14
	TFC (mg QE/g)	5.24 ± 0.02
	CT (mg/g)	4.01 ± 0.52
	Saponins (%)	27.55 ± 0.59
Antioxidant activity	DPPH EC_50_ μg/mL	63.30 ± 0.015
	FRAP (nmol/g)	15.02 ± 0.34
Ascorbic acid	μg/mL	0.87 ± 0.02

### 4.2 Biological evaluation of extract supplementation in vivo

The results of feed and water intake are presented in Tables 3 and 4. Maximum feed intake was observed in group 2 (30.99 ± 2.13 g) at the 4th week while the minimum feed intake was seen in group 4 (19.88 + 1.91 g) at the 3rd week, respectively.

**TABLE 3:** Feed intake (g).

Time period	Control	Experiential		
Weeks	Group 1	Group 2	Group 3	Group 4
1st week	21.04 ± 0.23^de^	23.55 ± 3.17^cd^	26.22 ± 2.17^bc^	24.55 ± 1.13^c^
2nd week	21.27 ± 0.25^de^	27.83 ± 1.86^b^	26.94 ± 1.10^b^	21.00 ± 0.5^de^
3rd week	22.05 ± 0.5^de^	30.77 ± 1.60^a^	25.44 ± 2.08b^c^	19.88± 1.91^e^
4th week	22.55 ± 0.94^de^	30.99 ± 2.13^a^	21.83 ± 0.60^de^	22.16 ± 2.07^de^

*Note*: Different superscript alphabets in the table are significantly different (*p* ≥ .05).

**TABLE 4:** Water intake (mL).

Weeks	Group 1	Group 2	Group 3	Group 4
1st week	18.92 ± 1.31^e^	22.00 ± 0.5^cd^	29.22 ± 0.34^a^	29.55 ± 0.25^a^
2nd week	22.05 ± 2.56^cd^	23.77 ± 1.07^c^	25.44 ± 0. 91^b^	24.16 ± 0.83^bc^
3rd week	22.66 ± 0.60^cd^	21.38 ± 0.19^d^	22.38 ± 0.55^cd^	21.38 ± 1.63^d^
4th week	23.11 ± 3.15^c^	25.00 ± 1.01^b^	21.22 ± 0.25^d^	20.05 ± 0.42^d^

*Note*: Different superscript alphabets in the table are significantly different (*p* ≥ .05).

The highest water intake (29.55 ± 0.25 mL) was observed in group 4 in the 1st week and the lowest water intake (20.05 ± 0.42 mL) was observed in the 4th week in the same group.

The body weight changes of the rat groups are shown in Table 5. According to the results, the highest body weight change was observed in group 4 while the lowest change was observed in group 2.

**TABLE 5:** Weight change (g).

Weeks	Group 1	Group 2	Group 3	Group 4
1st week	239.83 ± 10.33^c^	248.83 ± 9.49^b^	261.33 ± 10.05 ^a^	251.00 ± 13.92^b^
2nd week	215.00 ± 2.88^de^	224.33 ± 8.89^d^	233.00 ± 12.87^cd^	213.33 ± 9.22^e^
3rd week	206.17 ± 4.16^ef^	217.00b ± 6.44^de^	189.33 ± 13.10^f^	177.00 ± 12.40
4th week	182.50 ± 10.44^f^	204.50 ± 8.04^ef^	155.50 ± 6.56^g^	147.83 ± 6.9^g^

*Note*: Different superscript alphabets in the table are significantly different (*p* ≥ .05).

The values of lipids are presented in Table 6. Results have shown a significant (*p* = .00) decrease in TC, TG, and LDL of groups 3 and 4 as compared to group 2. HDL has increased significantly (*p* = .00) in groups 3 and 4 as compared to group 2.

**TABLE 6:** Effect of *C. fistula* extract on the lipid profile of hyperlipidemic rats.

Experimental groups	Lipid parameters (mg/dL)					*F* value
	Group 1	Group 2	Group 3	Group 4	*p* Value	
TC	73.33 ± 7.20	150.01 ± 6.16	115.33 ± 5.06	90.83 ± 8.86	.00**	131.26
TG	84.50 ± 7.36	119.67 ± 10.98	101.67 ± 9.33	74.16 ± 9.10	.00**	27.76
HDL	20.83 ± 2.99	17.33 ± 4.50	40.76 ± 4.38	41.83 ± 8.4	.00**	33.62
LDL	72.50 ± 6.53	114.00 ± 7.29	76.33 ± 4.32	74.83 ± 4.66	.00**	68.91

Table 7 shows the result of hepatic enzymes after intake of *C. fistula extract*. AST and ALT values were significantly (*p* = .00) reduced in groups 3 and 4 as compared to group 2.

**TABLE 7:** Effect of *C. fistula* extract on liver enzymes of hyperlipidemic rats.

Parameters	Control	Experimental groups				
	Group 1	Group 2	Group 3	Group 4	*p* Value	*F* value
AST (U/L)	57.083 ± 10.89	97.25 ± 12.93	73.00 ± 5.47	67.16 ± 10.60	.00**	16.31
ALT (U/L)	25.66 ± 3.50	56.50 ± 11.77	25.58 ± 4.56	22.33 ± 7.22	.00**	27.63

The hematological results are presented in Table 8. The HB, RBC, MCHC, and MCV were significantly (*p* = .00) higher in groups 3 and 4 as compared to group 2. Neutrophils, monocytes, and platelets were reduced significantly (*p* = .00) in both groups as compared to group 2.

**TABLE 8:** Effect on hematological parameters.

Parameters	Control	Experimental groups			Significance	
	Group 1	Group 2	Group 3	Group 4	*p* Value	*F* value
HB (g/dL)	11.045 ± 0.43	6.75 ± 0.80	11.06 ± 0.69	11.37 ± 0.43	.00**	81.37
RBCs (10^6^/μL)	8.145 ± 1.00	6.47 ± 0.36	7.81 ± 0.64	8.03 ± 0.67	.00**	6.94
MCHC (g/dL)	36.33 ± 3.93	23.67 ± 3.31	34.69 ± 0.62	35.02 ± 1.78	.00**	27.64
MCH (Pg)	17.31 ± 0.29	15.96 ± 2.41	17.29 ± 0.71	18.00 ± 0.26	.06	2.91
MCV (fL)	52.44 ± 3.49	42.52 ± 7.06	54.01 ± 1.77	55.36 ± 4.01	.00**	10.01
Neutrophil (%)	11.76 ± 2.32	36.35 ± 1.86	17.19 ± 8.55	11.23 ± 0.51	.00**	40.43
Monocytes (%)	2.033 ± 0.85	4.38 ± 0.21	2.13 ± 1.12	2.01 ± 0.63	.00**	12.81
Platelet (10^3^/μL)	848.67 ± 96.61	990.33 ± 90.03	765.67 ± 57.78	755.00 ± 32.38	.00**	12.60

**FIGURE 1:**
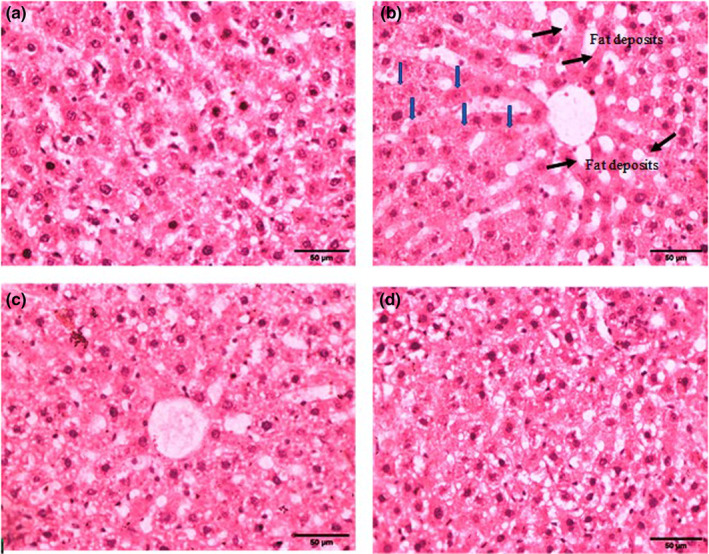
Histopathology images of liver at 40× magnification fixed with 10% formalin and stained with hematoxylin for fatty liver analysis. The fat accumulation in black arrows and necrosis in blue arrows induced by HFD‐induced hyperlipidic rats are shown in (b) while the protective effect of *C. fistula* extract in HFD‐induced hyperlipidic rats is shown in (c and d). (a) is non‐HFD‐induced untreated control group.

Figure 1 illustrates the histopathological changes in the liver. The extension of a pathological change as rated ‘score value’ and the pathological importance of this alteration as ‘importance factor’ were collectively used to categorize the changes. According to the score, architectural and structural alteration with fat deposits and necrosis was observed. Histopathology images of fatty liver showed fat accumulation as black arrows and necrosis as blue arrows induced by HFD‐induced hyperlipidic rats in Figure 1b. However, fat accumulation and inflammatory necrosis reduction in dose‐dependent manner were observed in HFD‐induced hyperlipidic rats shown in Figure 1c,d which indicates the protective effect of *C. fistula* extract. Figure 1a is non‐HFD‐induced untreated control group showing normal structure of liver histology.

## 5 DISCUSSION

The proximal composition analysis of pods aligned with prior studies (Kdam, 2022; Shukla et al., 2013), yet TPC and TFC were notably higher than reported values by previous studies (Chourasia et al., 2019) and potentially attributed to variations in plant part's utilization. Comparisons with the results of Irshad et al. (2012) revealed discrepancies in TPC and TFC levels, possibly due to differences in extracting solvents and plant part's utilization. Such variations in phytochemical composition across studies may stem from various geographic regions and variations in plant nutrition. Our assessments encompassed yield extraction, proximal analysis, phytochemical content, and antioxidant activity showed that notably water and UAE extraction methods yield superior results compared to methanol/ethanol extraction and consistent with findings by Tan et al. (2018). Assessment of antioxidant activity revealed promising results, consistent with prior findings by Abdellatif et al. (2024). These results suggest that *C. fistula* extract possesses potent antioxidant properties, attributed to specific phytochemical constituents. Observations on experimental groups indicated significant variations in feed intake over time, potentially influenced by hyperlipidemia effects. Notably, reductions in feed intake correlated with *C. fistula* extract consumption, consistent with reports by Shah et al. (2019). Distinct patterns of water intake, with higher initial levels followed by gradual reductions, may reflect metabolic differences and dietary fiber content, as observed in hyperlipidemic rats (Tamargo et al., 2020). Significant reductions in body weight among hyperlipidemic groups suggest metabolic alterations associated with *C. fistula* extract consumption, supporting previous studies indicating its role in suppressing cholesterol and TG biosynthesis (Abid et al., 2016; Jangir & Jain, 2017).

The efficiency of *C. fistula* extract in modulating lipid metabolism and exerting antioxidant effects elucidated its potential mechanisms underlying the observed effects, attributing reductions in LDL levels to enhanced hepatic LDL receptor binding facilitated by polyphenolic extracts (Kaur et al., 2019). Moreover, increases in HDL levels may result from enhanced lecithin‐cholesterol acyltransferase activity, promoting reverse cholesterol transport and endothelial protection (Ossoli et al., 2016). *C. fistula*'s hepatoprotogenic effects were evidenced by decreased AST and ALT levels, attributed to its antioxidant compounds, consistent with reports by Kaur et al. (2019). These findings underscore the potential of *C. fistula* extract as a hepatoprotogerative agent. The study highlighted immunomodulatory effects, with flavonoids such as quercetin enhancing erythropoietin activity, leading to elevated neutrophil, monocyte, and platelet levels in hyperlipidemic rats. Notably, *C. fistula* extract mitigated these effects, suggesting its potential in ameliorating oxidative stress‐induced hematological alterations (Abdellatif et al., 2024; Straface et al., 2010). While reductions in neutrophil and monocyte levels attributed to flavonoid extracts, align with prior studies indicating their role in mitigating stress‐induced immune responses (Lin et al., 2022). Moreover, decreases in platelet counts suggest potential antiplatelet effects, indicative of vascular disease prevention (Asgary et al., 2012). Histopathological examination provides valuable insights into the structural alteration induced by hyperlipidemia and the protective effects of *C. fistula* extract. Histological analysis of liver tissue revealed lipid accumulation, hepatocellular injury, and inflammatory infiltrates as necrosis characteristic of hyperlipidemic conditions. The current study's histopathological findings may exhibit improvements in liver architecture following *C. fistula* extract administration, characterized by reduced lipid accumulation, hepatocellular, and inflammatory changes as necrosis. These observations are consistent with previous reports indicating the hepatoprotective effects of *C. fistula* against induced liver damage (Thompson et al., 2017). The antioxidant and hepatoprotective effects underscore the consistent pharmacological potential of C. fistula and can be used as lipid‐lowering agents in hyperlipidemia.

## 6 CONCLUSION

The outcome of the current study demonstrated that *C. fistula* extract is an excellent source of polyphenol compound and has strong antioxidant potential that can provide probable benefits to consumers. Results of the efficacy study have shown anti‐hyperlipidemic effects of extract of *C. fistula*. It decreases TC, TG, LDL, and HDL in hyperlipidemic rats.

## AUTHOR CONTRIBUTIONS

**Maryam Tariq:** Conceptualization (equal); data curation (equal). **Nazir Ahmad:** Project administration (equal); supervision (equal); writing – original draft (equal). **Mahr Un Nisa:** Validation (equal). **Muhammad Abdul Rahim:** Funding acquisition (equal); writing – review and editing (equal). **Eliasse Zongo:** Funding acquisition (equal); writing – review and editing (equal).

## FUNDING INFORMATION

No specific grant was used in this study.

## CONFLICT OF INTEREST STATEMENT

No competing interests for any purpose are declared by the authors.

## ETHICAL APPROVAL

Government College University Faisalabad intuitional ethical approval was obtained from the ethic review committee under registration number Ref. No. GCUF.ERC/23.

## Data Availability

It is original and unpublished research data and will be available as per publisher policy.
